# Faith-Placed Services and Supports for Alzheimer’s disease and related dementias: A Scoping Review

**DOI:** 10.1093/geroni/igaf122.3932

**Published:** 2025-12-31

**Authors:** Noelle Fields, Ling Xu, Mary Stringfellow, Katherine Kitchens, Soeun Jang

**Affiliations:** The University of Texas at Arlington, Arlington, Texas, United States; University of Texas at Arlington, Arlington, Texas, United States; UT Southwestern Medical School, Dallas, Texas, United States; UT Southwestern Medical Center, Dallas, Texas, United States; University of Texas at Arlington, The University of Texas at Arlington, Texas, United States

## Abstract

Faith-based communities, including churches, temples, and other places of worship, have been identified as important sources of support for Alzheimer’s disease and related dementias (AD/ADRD) family caregivers. The role of faith communities as important sources of support has been deeply rooted in cultural, religious, and historical contexts. However, the extent and nature of services and supports provided in faith-placed settings remain unclear. This scoping review focused on the existing literature on faith-placed interventions and services that support AD/ADRD family caregivers. Following Arksey and O’Malley’s (2005) scoping review framework, we searched seven databases, including Academic Search Complete, CINAHL Complete, and PsycINFO, for peer-reviewed studies published through August 2024. Studies were eligible if they examined services, interventions, or supports for AD/ADRD family caregivers in places of worship. Two independent reviewers screened articles, and data were charted and thematically analyzed. Eleven studies met inclusion criteria. Faith-placed supports varied widely, including dementia education, caregiver support groups, respite care, and dementia-friendly worship services. These interventions were particularly impactful in underserved and rural communities. Common themes included increased dementia knowledge, emotional and social support, and dementia-friendly modifications. Barriers to implementation included limited clergy training, resource constraints, and cultural stigma around dementia. Faith-placed settings play a critical role in supporting AD/ADRD caregivers.The geographical context of the studies may play an important role in shaping the design and effectiveness of faith-placed interventions for AD/ADRD. Barriers to faith-placed interventions remain, highlighting the need for continued refinement and expansion of such programs.

